# Vivax malaria: a possible stumbling block for malaria elimination in India

**DOI:** 10.3389/fpubh.2023.1228217

**Published:** 2024-01-08

**Authors:** Ashwani Kumar, Puspendra Pal Singh, Suchi Tyagi, K. Hari Kishan Raju, Sudhanshu S. Sahu, Manju Rahi

**Affiliations:** ^1^ICMR - Vector Control Research Centre, Puducherry, India; ^2^Indian Council of Medical Research, Hqrs New Delhi, India

**Keywords:** *Plasmodium vivax*, relapse, hypnozoites, glucose-6-phosphate dehydrogenase, primaquine, vaccine, elimination

## Abstract

*Plasmodium vivax* is geographically the most widely dispersed human malaria parasite species. It has shown resilience and a great deal of adaptability. Genomic studies suggest that *P. vivax* originated from Asia or Africa and moved to the rest of the world. Although *P. vivax* is evolutionarily an older species than *Plasmodium falciparum*, its biology, transmission, pathology, and control still require better elucidation. *P. vivax* poses problems for malaria elimination because of the ability of a single primary infection to produce multiple relapses over months and years. *P. vivax* malaria elimination program needs early diagnosis, and prompt and complete radical treatment, which is challenging, to simultaneously exterminate the circulating parasites and dormant hypnozoites lodged in the hepatocytes of the host liver. As prompt surveillance and effective treatments are rolled out, preventing primaquine toxicity in the patients having glucose-6-phosphate dehydrogenase (G6PD) deficiency should be a priority for the *vivax* elimination program. This review sheds light on the burden of *P. vivax*, changing epidemiological patterns, the hurdles in elimination efforts, and the essential tools needed not just in India but globally. These tools encompass innovative treatments for eliminating dormant parasites, coping with evolving drug resistance, and the development of potential vaccines against the parasite.

## 1 Introduction

*Plasmodium vivax* is historically a resilient and the most widely distributed malaria parasite species. It coexists with *Plasmodium falciparum* in different proportions and geographical regions, and infrequently mixed infection of both species is also encountered (1, 2). *P. vivax* is known to cause extensive morbidity and low incidence of mortality across the world (3). Africa has the highest burden of malaria but mostly of *P. falciparum.* The endemic countries outside Africa have a sizable burden of *P. vivax* clinical malaria (4). The WHO Southeast Asia Region globally accounts for 53% of the *P. vivax* burden, with India accounting for the majority (i.e., 47%) in 2018 (5). In India, the proportion of *P. vivax* and *P. falciparum* malaria has been equal in the last two decades, although in recent years, vivax malaria has shown fluctuating trends being 38% in 2017 (6), 52% in 2018, 54% in 2019 and 36% in 2020 (7). Vivax malaria has certain distinctive characteristics that make it highly problematic such as multiple strains of *P. vivax* (Table 1) which are characterized by the pattern of relapses and duration of latency (8, 9). Jennison *et al* reported that *P. vivax* populations are more genetically diverse than *P. falciparum*, indicating greater resilience to environmental challenges and higher levels of interbreeding within and between distant parasite populations (10). *P. vivax’s* unique characteristics, such as relapse, provide opportunities for the exchange and dissemination of genetic material (11).The rapid development of immunity and high transmission potential of *P. vivax* is a unique characteristic of this malaria parasite and presents significant challenges to malaria control and elimination efforts. The species has a shorter growth phase in vectors, produces hypnozoites, and makes gametocytes earlier to the development of clinical manifestations in the patients, which allows the gametocytes to be picked up by the mosquito before the patient seeks treatment. These features and adaptabilities provide an edge to *P. vivax* to maintain a steady transmission via seasonal mosquito vectors, even at lower ambient temperatures and proves to be a major challenge in controlling transmission (12). Further, due to the prolonged latency in the liver phase, *P. vivax* ensures survival in colder regions, making it geographically the most widely distributed malaria parasite species capable of persisting in tropics, subtropics, and temperate climates (13). Children show greater morbidity in *P. vivax* endemic areas than adults who frequently have asymptomatic infections (14).

**Table 1:** The list of different strains of *P. vivax* based on the latency period.

S. No.	*Plasmodium vivax* strains	Period of latency
1	Chesson strain	Short period of latency (regular intervals of 3 to 4 weeks more than 5 months)
2	St Elizabeth strain	Long period of latency (up to 2 years)
3	Netherlands strain	Long period of latency (8 months)
4	North Korean strain	Long period of latency (8–11 months)
5	*Sal 1* strain	Long Latency
6	Madagascar strain	Long period of latency (8–13 months)
7	McCoy strains	Long period of latency
8	North Indian strain	Long period of latency (8–13 months)

The global incidence of malaria has declined in the past 15 years, largely due to *P. falciparum* control. Malaria incidence (cases per 1,000 people at risk) fell from 81 in 2000 to 59 in 2015, and 56 in 2019 before rising to 59 in 2020. The increase in 2020 was mainly linked to the disruption in the surveillance and vector control services caused by the COVID-19 pandemic. Similar gains have been elusive in the case of *P. vivax* as it is less amenable to the routine malaria control interventions designed against falciparum malaria (15). Because *P. vivax* was inappropriately perceived as a benign infection, control of *P. vivax* has made slower progress relative to *P. falciparum* since 1960 (16). Justifiably, global malaria elimination efforts are focused against *P. falciparum* due to extensive morbidity and mortality caused by it in Africa and forested areas inhabited by ethnic tribes in Asia, particularly in India. The focus is more so on children and pregnant women, and therefore more resources are available for the control of *P. falciparum* than *P. vivax,* even though both species are generally co-endemic, especially in the Southeast Asia Region. It is a prevalent view among malariologists that eradicating *P. vivax* is technically more cumbersome than eliminating *P. falciparum* due to knowledge gaps and fewer reliable tools, especially for the prevention of recurring relapses (12, 16).

## 2 Global burden of *Plasmodium vivax*

**Figure 1:**
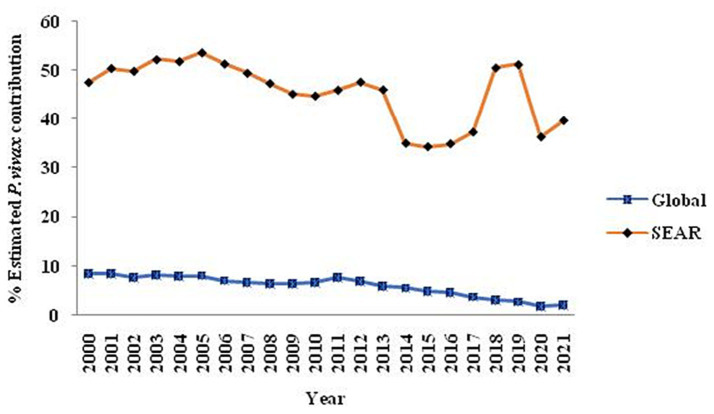
The trend over 20 years regarding the contribution of *P. vivax* globally and within the Southeast Asia Region is illustrated in a graph extracted from the WHO-2021 report, specifically on pages 23 and 28.

Globally, *P. vivax* and *P. falciparum* account for most human malaria infections. Though *P. falciparum* is known for significant global morbidity and mortality, *P. vivax* is common among all human *Plasmodia,* and despite being benign, it also causes severe and even fatal infections (13, 17). *P. vivax* infection threatens one-third of the world’s population (~ 2.5 billion people) (17). In the 2021 WHO Report, there were ~ 241 million estimated malaria cases accountable for about 627,000 deaths; among them, 2.0% were attributed to *P. vivax*. In 2020, *P. vivax* accounted for roughly 2% of the worldwide malaria burden, marking a notable decline from its previous contribution of nearly 8% in 2000. The vivax burden was also reduced in WHO South-East Asian region from 47.7% (2000) to 36.3% (2020) (Figure 1). In 2020, India was responsible for nearly 83 percent of all malaria infections, with *P. vivax* accounting for more than a third (39%) of all cases in the South-East Asia region (18).

## 3 *Plasmodium vivax* burden in India

**Figure 2:**
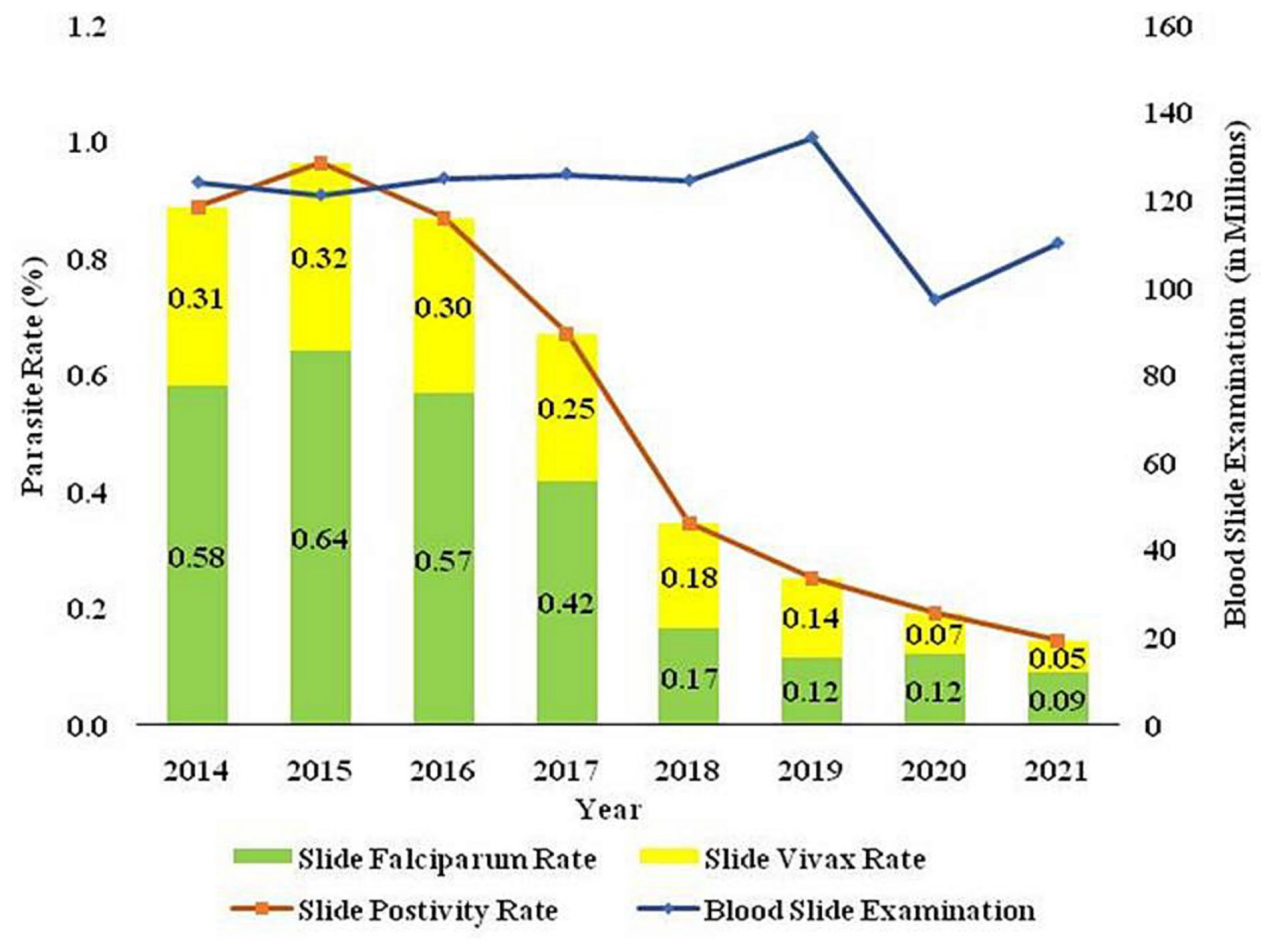
The trend of blood smears examined (BSE), Slide Positivity Rate (SPR), Slide falciparum rate (SFR), and Slide vivax rate (SVR) over the time in India is based on data sourced from the National Centre for Vector Borne Disease Control (NCVBDC), India.

**Figure 3:**
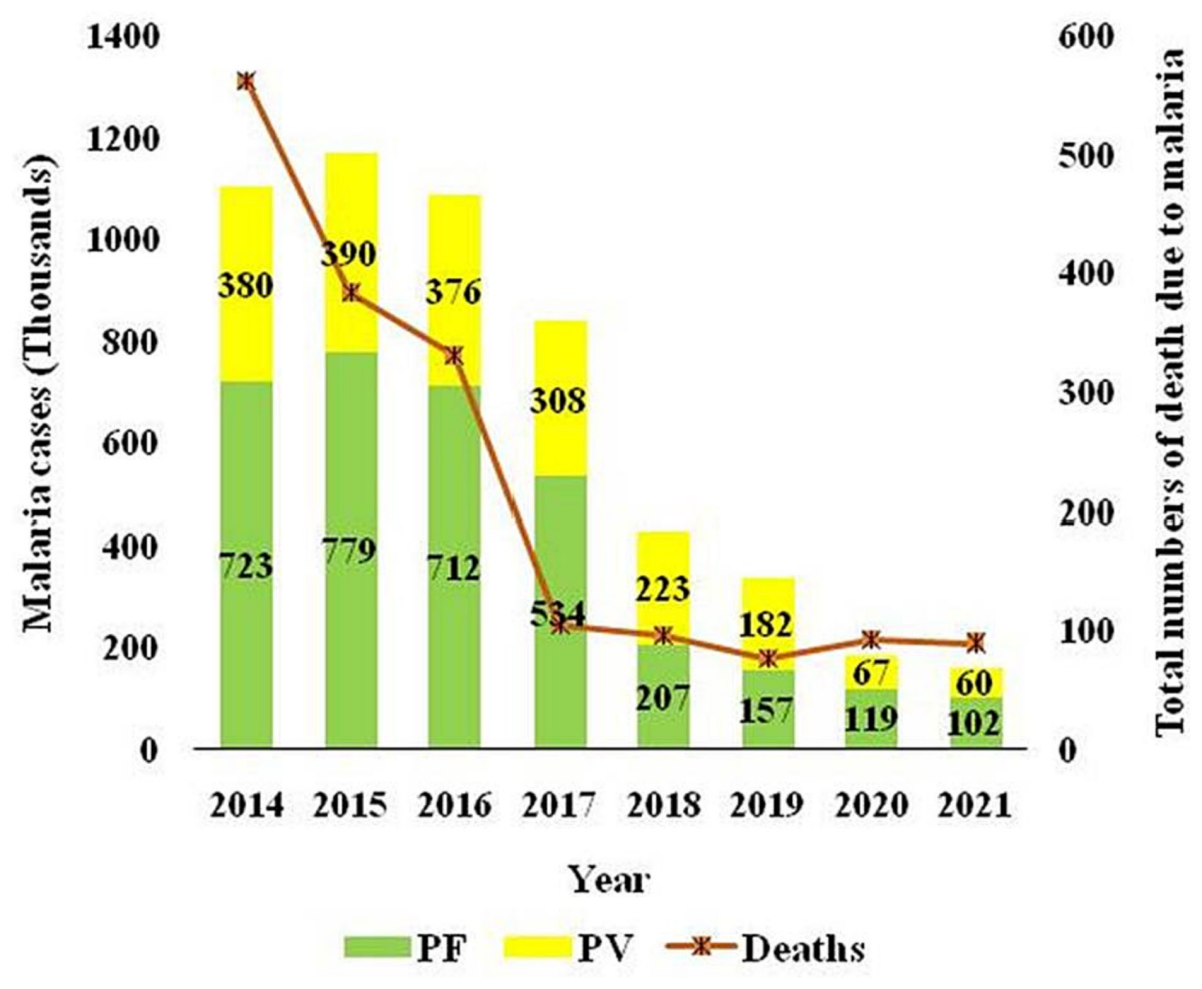
The trajectory of reported cases attributed to *P. vivax* and *P. falciparum* and total deaths due to malaria over the time in India is sourced from the National Centre for Vector Borne Disease Control, India.

**Figure 4:**
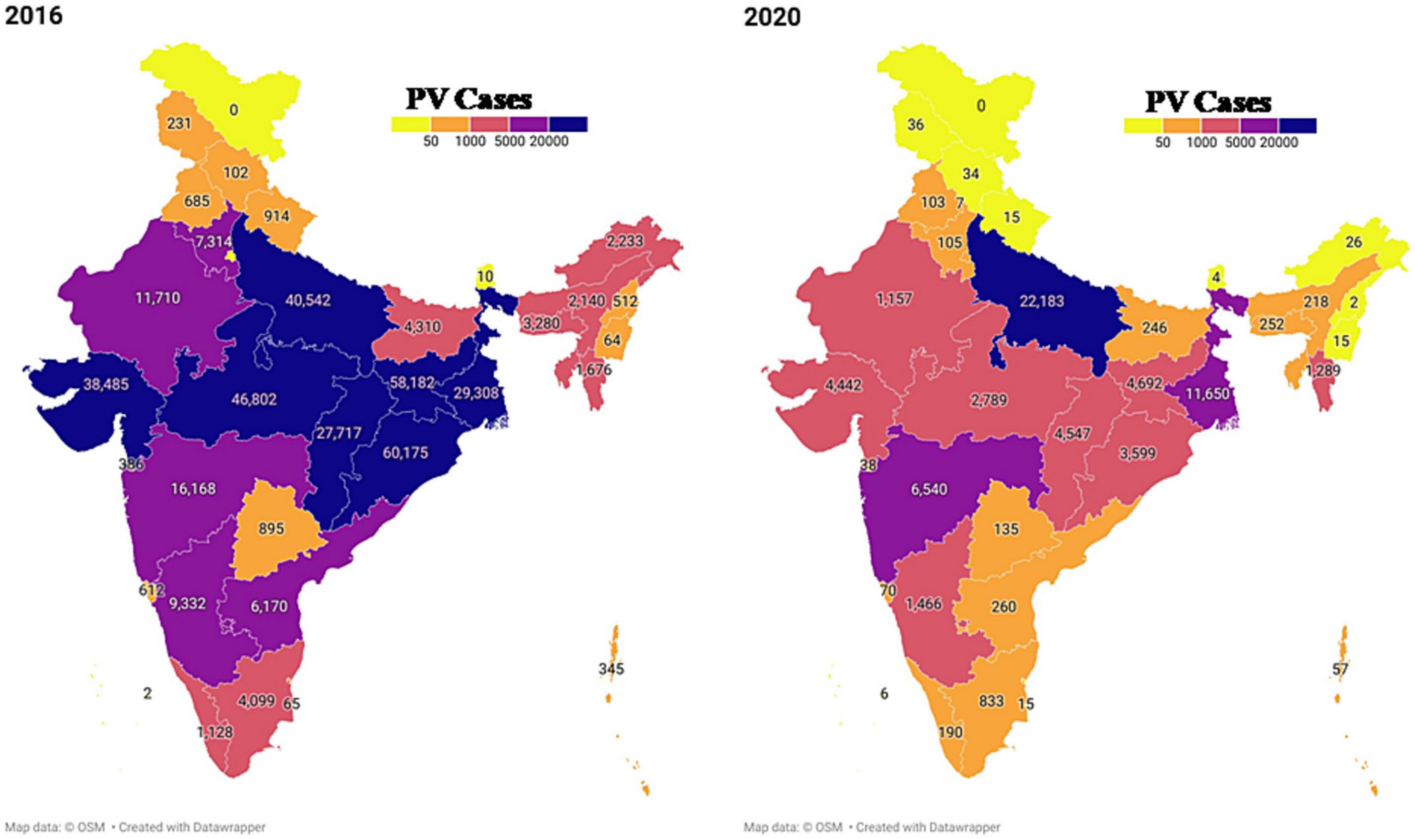
The geographical spread of *P. vivax* across India is depicted using data reported by NCVBDC for the years 2014 and 2020. The map was generated utilizing the available NCVBDC data through the data wrapper software.

The two major malaria parasites in India are *P. falciparum* and *P. vivax*. *P. vivax* is mainly found in plains, but *P. falciparum* is present in woodland and bordering areas (19). India has a robust surveillance system that consistently collects and reports data. Over 100 million blood slides are examined in India from 2014–2021, making it an excellent source of data from all endemic districts. The slide positivity rate (SPR) is decreased from 0.89% in 2014 to 0.14% in 2021. The slide positive rate was higher in *P. falciparum* (0.09%) than *P. vivax* (0.05%) in the same period (Figure 2). As per the national malaria control program, vivax malaria declined to 37.3% in 2020 from 53.63% in 2019 (Figure 3). During 2021, *P. vivax* accounted for around ~38% (59087) of all malaria cases (158326) reported in the country (6). A surveillance-based national burden study conducted in 2015 and 2016 had estimated 1.08 million (28%) cases of *P. vivax* out of 3.875 million total malaria cases and 572 (3.0%) deaths out of 19,067 confirmed deaths attributed to the species in India, while the remaining cases and deaths were due to *P. falciparum* (20). The incidence of *P. vivax* and *P. falciparum* was almost equal nationally for nearly 15 years (1999–2014), probably due to chloroquine resistance against falciparum malaria (21). With the introduction of Artemisinin-based combination treatments (ACTs) for treatment of falciparum malaria in 2007, a decline in falciparum and overall malaria cases was observed. By 2014, the *P. vivax* proportion declined to 34%, with a high regional variation, which might be due to better management of both *Plasmodium* species. In 2014, about 380,000 *P. vivax* cases were recorded in India; which was approximately 16.6% of all *P. vivax* cases reported globally in 2014. Eleven out of 29 states in India accounted for around 95% of *P. vivax* cases, and of these 75% of cases were reported from seven states *viz.*, Maharashtra, Madhya Pradesh, Jharkhand, Odisha, Gujarat, Chhattisgarh, and Uttar Pradesh. *P. vivax* has a much higher prevalence than *P. falciparum* in the urban areas of India (22) (Figure 4). Although reported incidence and mortality of malaria has declined due to the recent malaria elimination drive in India, in most states, it was largely due to the successful control of *P. falciparum* (23). In northeastern India, *P. vivax* is still a neglected infectious disease. Estimating recurrence patterns and transmission dynamics of *P. vivax* in different ecological settings are important prerequisites for malaria elimination programs in northeastern states (24).

## 4 Origin and dispersal of *Plasmodium vivax*

The origin and migration pattern of the malaria parasite, *P. vivax,* has always been a matter of debate. Understanding the origin, population migration pattern, and genetic diversity is important for improving strategies to fight against the malaria parasites and avert further spread. The routes of population migration and the origin of the parasites can be inferred through the evolutionary genomics approach adopted by evolutionary biologists (25).

The two evidence-based scenarios mainly discussed are whether this species originated in Asia or Africa. Based on 106 mitochondrial genome sequences from nine different countries, it was hypothesized that *P. vivax* had adapted itself, if not jumped from monkeys to humans, in South East Asia, and the species subsequently expanded in Asia and might have migrated to Africa through human population movements across the continents (25). Another study using the mitochondrial genome has revealed lesser genetic diversity in extant African *P. vivax* in comparison to the rest of the world and suggested a recent introduction in Africa from the Indian subcontinent (26). The Asian origin is further supported by one of the migration models utilizing 941 global mitogenomes of *P. vivax* (27). Recently, a study covering 28 locations in 20 countries reported the highest microsatellite genetic diversity in *P. vivax* populations in Southeast Asia and advocated the Asian origin of *P. vivax* (28).

With the discovery of *P. vivax*-like and several other parasites in African apes like chimpanzees and gorillas, it is now being hypothesized that *P. vivax* originally might have passed on from apes to humans in Africa instead of Asia (29, 30). A total of 5,000 ape faecal samples from 78 remote forest sites were screened for *Plasmodium* species throughout central Africa. It was an interesting discovery that the infection rates of *P. vivax*- like parasites were the same in the wild ape communities and in human populations, with stable parasite transmission (29). Considering that the above study was based on a few genes and partial genomes from the mitochondria, the observed phylogenetic patterns may be one-sided due to incomplete lineage sorting. This hypothesis is given further credence by the *P. vivax* parasite’s ability to infect Duffy-negative humans in Africa and South America (31–34). Recently, Van Dorp et al. (35) proposed an additional route of migration of *P. vivax* from Africa to Europe and then to South America using old blood smears of malaria patients who had malaria between 1942 and 1944 in the Ebro Delta of Spain. They have suggested utilizing historic medical collections for retrieving genomic information to infer the exact evolutionary history of the malaria parasite species.

The high genetic diversity of Asian *P. vivax* is an important factor to consider while suggesting the origin of the species. In addition, the phylogenetic closeness of *P. vivax* with fifteen *Plasmodium* species infecting wild Asian monkeys also needs to be considered. Recently, the discovery of the human malaria parasite, *P. falciparum,* in monkey species *Macaca mulatta* and *M. Radiate* (36) and non-human primates (*P. coatneyi* and *P. fragile*) specific variation reported in human *P. falciparum* in India (37). The non-human malaria parasite *Plasmodium knowlesi* has been observed in both human hosts and vectors across various regions in India, including Bihar, Delhi, Andaman & Nicobar Islands, and Uttar Pradesh, it’s crucial to base conclusions on robust scientific evidence (38–41). Additional evolutionary studies on malaria parasites of Asian monkeys will provide conclusive evidence to settle the debate on the origin of species from the academic point of view. To that effect, it is suggested to sequence the whole genome of *P. vivax* from worldwide populations to pinpoint the origin and dispersal pattern of the species.

## 5 Relapse of *Plasmodium vivax*

**Figure 5:**
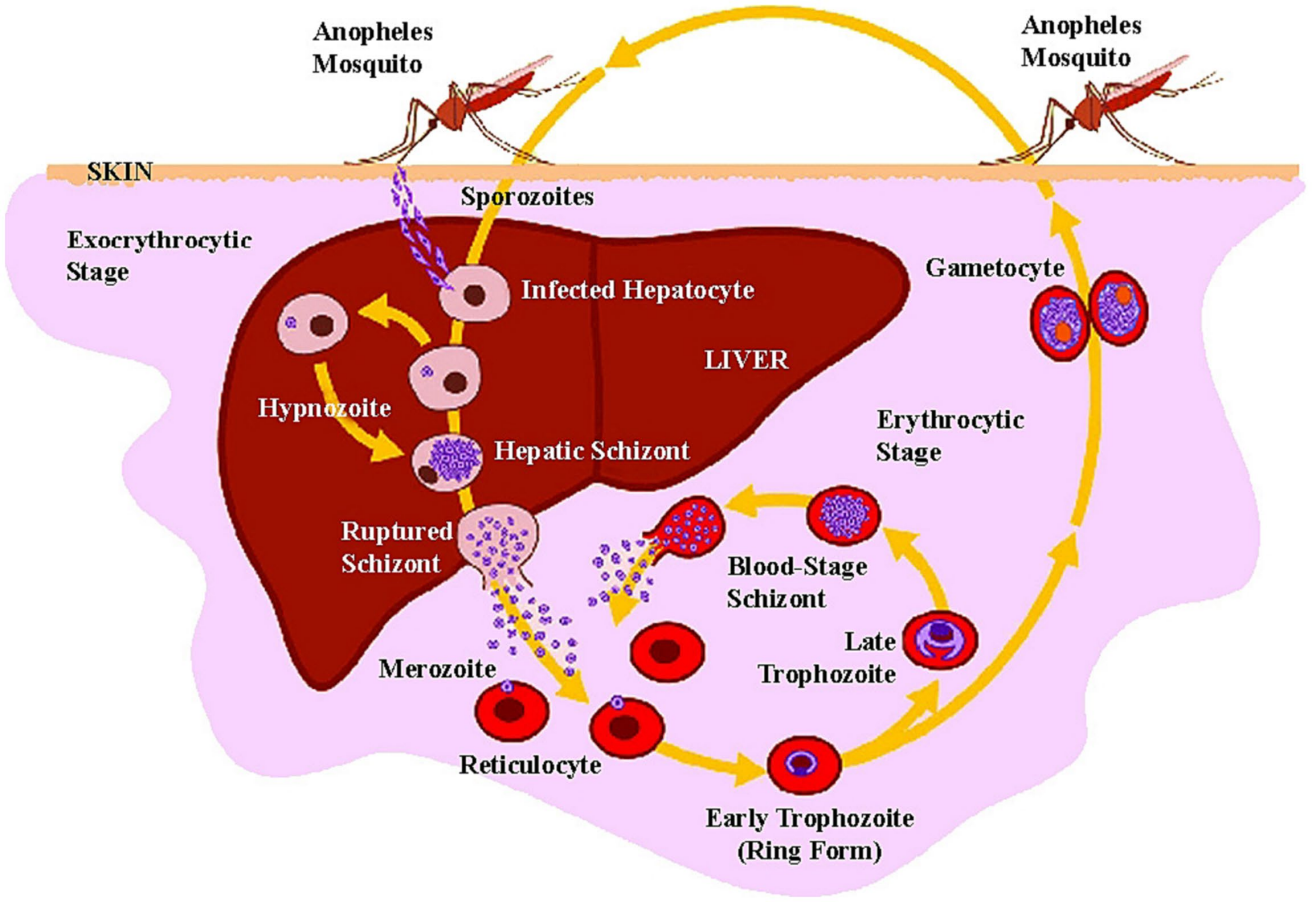
Life cycle of *P. vivax* in human host (adapted from “Major Histocompatibility Complex and Malaria: Focus on *P. vivax* Infection” by Lima-Junior JC and Pratt-Riccio LR (2016). Front. Immunol. 7:13. Page no-4. Copyright © 2016 Lima-Junior and Pratt-Riccio).

One *P. vivax* infective bite of a mosquito vector results in an attack within 2 weeks. It may be followed by multiple clinical episodes ranging from 2 months to 4 years after the initial infection (17). *P. vivax* produces dormant stages in the liver called hypnozoites, which, without any signs, become active and re-infect the bloodstream and trigger fresh episode of vivax malaria (Figure 5) which is the main setback for any malaria elimination program.

To target the pattern of relapse and prevent active transmission, different *P. vivax* strains in various parts of the world would require specific intervention strategies. Therefore, knowledge of the pattern of relapse is highly necessary to make an effective strategy for *P. vivax* elimination. It has been speculated that the relapse latency in different parasite populations depends on the type of strain, host biology, and climatic conditions (17). Variation in relapse patterns has been well studied in India. The strains with differing patterns of relapse coexisted, resulting in complications in transmission control measures. *P. vivax* populations have been identified as polymorphic for relapse in Delhi, India *viz.*, Tropical type (those relapsing between 1 and 3 months); Sub-tropical types (relapsing between 12 and 20 weeks) and temperate type (relapsing between 24 and 28 weeks) (42, 43).

Globally, eight strains of *P. vivax* have been recognized (Table 1) *viz.*, Chesson strain (with short latency and repeated relapses at short intervals), St. Elizabeth strain (long latency up to 2 years), Netherlands strain (long latency of ~8 months), North Korean strain (long latency of 8–11 months), sal 1 strain (long latency), Madagascar strain (long latency of 8–13 months), McCoy strain (long latency) and North Indian strain (long period of latency of 8–13 months) (8, 9). It should be noted that strains that relapse quickly are thought to originate in Southeast Asia. Long-latency strains are found in temperate and subtropical areas (9). Long-latency and frequently relapsing strains exist in the Indian subcontinent and South America (9). The dormancy of hypnozoites is usually longer in temperate zones, where vectors are active only for a few months during favourable weather (44).

It is still unclear what specific triggers are required for hypnozoite activation and reactivation though bacterial infections and infection with falciparum malaria are incriminated. Normal parasites and bacteriological infections can trigger hypnozoites of *P. vivax* (45).

## 6 Clinical presentation and complications

Pathogenesis of *P. vivax* malaria is consistent with RBC rupture in mature schizonts. In the early stages of *P. vivax* infection, fevers may be intermittent, and symptoms may be atypical, but if left untreated, these become intermittent (every other day) and typical. It causes the first appearance with hiccups (46), lack of fever sometimes, loss of taste, cough, pain while swallowing, and urinary discomfort (47). Infection may also lead to splenomegaly (48).

*P. vivax* is traditionally considered a benign species causes mild, uncomplicated, and self-limited illness (49). The public health value of controlling aggressive *P. falciparum* outshines *P. vivax, as* falciparum malaria leads to mortality due to complications and involvement of multiple organs. However, vivax malaria has also recently caused life-threatening complications, especially among children and pregnant women in endemic countries including India, Brazil, and Indonesia (49–54). Some severe malaria complications that have been reported include cerebral malaria, organ dysfunction, jaundice, acute kidney injury, thrombocytopenia, hypoglycemia, hepatic dysfunction, renal impairment, and hypotension (55–59). These observed symptoms could be due to arbitrary use of anti-malarial drugs, delayed treatment, increased resistance, and the change in the clinical spectrum of the disease (53, 55).

There are established reports that *P. vivax* causes severe anaemia among children in vivax-endemic countries (3, 49). It is well known that, unlike *P. falciparum*, the *P. vivax* merozoites prefer reticulocytes. The other causes of severe anaemia may be due to the release of glycosylphosphatidyl-inositol toxin in non-infected erythrocytes, the intensive haemolysis of circulating infected RBCs, other inducers of irritation such as haemozoin and dyserythropoiesis that occur due to the effect of different cytokines (60–62). A ruinous case of *P. vivax* malaria was reported in 2018 from the state of Goa, India. A 20-year-old otherwise healthy woman was diagnosed with vivax malaria with high parasitaemia (140,000/μl), confirmed microscopically followed by Polymerase Chain Reaction. She suffered from acute respiratory distress (ARDS) and succumbed to the so-called benign vivax malaria. Post-mortem showed congestion of alveolar capillaries, heavy monocytic infiltration and damage to the alveolar membranes consistent with ARDS (63).

## 7 Diagnosis of *Plasmodium vivax*

Currently, two diagnostic methods for malaria parasites in the point of care or clinical setting, i.e., Microscopy and rapid diagnostic tests based on lactate dehydrogenase (LDH) enzyme, are frequently used. Polymerase chain reaction (PCR) and other molecular tools are seemingly more feasible in the research facilities to confirm malaria species and to study intra-specific variations at the genomic level. Lately, their utility is also being recognized for surveillance in programmatic settings in certain scenarios like persistent malaria (64).

There are two biological characteristics of *P. vivax*, which make its detection very difficult. Firstly, the parasite prefers to attack young red blood cells (reticulocytes) in the blood flow resulting in lesser parasitaemia, thus requiring high microscopic abilities for accurate diagnosis. The second significant problem is the dormant liver stages of hypnozoites, when activated, can cause multiple “relapse infections” with low initial parasitaemia. When at low densities, the parasite may not be easily detectable in blood smears with light microscopy.

The age-old light microscopy for direct visualization of *P. vivax* parasites is hailed as the gold standard for identification of *vivax* malaria and calculating parasitaemia (65). Since the 1990s, point-of-care RDTs have become increasingly popular diagnostic tools (65). RDTs can detect *Plasmodium* antigens in the circulating blood. However, the experience shows that the sensitivity of RDT drops at parasite densities less than 500/microliter blood for *P. falciparum* and *vivax* which may produce false negative results, restricting their use in *P. vivax* diagnosis (66). The falciparum diagnostic protein (HRP-2) is absent in vivax malaria, which is the basis of malaria rapid detection tests (67). As a result, the RDT used for vivax identification depends on lactic acid dehydrogenase, which is less sensitive than HRP-2-based tests for falciparum malaria (68–70). However, the national program does not track malaria cases caused by *P. malariae (Pm), P.ovale (Po)* and *P. knowlesi (Pk)*. Data on these non-*PfPv* infections are typically available only through published case reports and other studies, often as secondary outcomes (71–74). Chaturvedi et al. (41) reported that *P. malariae* and *P.ovale* infections, while less prevalent than *P. vivax* and *P. falciparun*, have been reported in India since 1930 and the 1980s, respectively. *Plasmodium knowlesi* infections were reported from 2004 onwards. *Plasmodium malariae*, in particular, is shown to persist in a single host for decades, even in the absence of symptoms, which provides an opportunity for sustained transmission. The study identifies potential hotspots for these neglected *Plasmodium* species, with Odisha and Bastar Chhattisgarh potentially being hotspots for *Plasmodium malariae* and *Plasmodium ovale* infections, respectively. Human *Plasmodium knowlesi* infections are more common in neighboring Southeast Asian countries like Malaysia, with only two studies reporting *Plasmodium knowlesi* infections in India. While existing anti-malarial drugs can be used to treat non-*PfPv* or mixed infections, the continued presence of these neglected parasites in the population, which often go undiagnosed, may pose challenges in achieving the goal of malaria elimination by 2030. Due to the lack of comprehensive data on overlooked (non-*PfPv*) infections in India and the pressing requirements for tailored treatments, disease prevention, an overall understanding of disease epidemiology, management of transmission dynamics, and the development of effective malaria control strategies is vital to integrate their surveillance and diagnosis within the national program. Hence, it is now required to develop improved diagnostic tools suited to low parasitaemia of non-falciparum species and probably non-LDH based (75). It is hopefully expected that the new technologies will eventually change this century-old diagnostic paradigm. However, it has been elusive despite the ongoing improvement in immuno-chromatographic strip tests. At the same time, proteomic techniques began to facilitate the identification of specific antigens of *P. vivax* (76). Discovering host responses, novel parasite antigens, and immune signatures using proteomic techniques can fill the diagnostic gap for *P. vivax*, which has proven difficult when parasitaemia is below the detection limit microscopic, and would play a vital role in global malaria elimination efforts (76). Based on serological markers and nucleic acid amplification techniques (NAATs), alternative diagnostic methods are also emerging nowadays (77). However, due to the high cost and advanced technology of NAAT-based approaches, their use in diagnosis is currently very limited.

## 8 Treatment

The treatment of *P. vivax* needs a combination of two antimalarials, a scizontocidal drug (4-aminoquinoline) to clear circulating stages and a hypnozoitocidal drug (8-aminoquinoline) for targeting hypnozoites in the liver. Chloroquine and primaquine are the only recommended drugs for treating *P. vivax* malaria, as per WHO guidelines. Confirmed cases of *P. vivax* has cured from the chloroquine with the dosage of 25 mg/kg for 3 days to remove an infection from the bloodstream, and primaquine with the dosage of 0.25 mg/kg for 14 days to cure hypnozoites, referred radical treatment. The treatment compliance of *P. vivax* is a challenge because of the 14 days treatment regimen with primaquine.

Primaquine is, however, contraindicated in pregnant women, infants, and persons with the deficiency of glucose-6-phosphate dehydrogenase (G6PD) (78–80). Because of the risk of foetal-haemolysis, it is strongly not recommended for pregnant women (79). This drug is also contraindicated in persons with met-haemoglobin reductase deficiency (79). Hence, individuals with G6PD deficiency must be recognized to evade drug-induced haemolysis prior to using primaquine (80). The adherence data of primaquine therapy is lacking (81). The want of access to radical treatment and poor adherence to a 14 days regimen will continuously fuel the number of *P. vivax* infections because of the many relapses which continue to cause *P. vivax* transmission. The development of drug resistance in *P. vivax* malaria is much less owing to its hypnozoite reservoir that maintains a pool of parasites drawn away from the bloodstream and maintained in the liver and protected from the drug effect because of their metabolic inactivity (82).

## 9 Improved drugs for control/elimination of *Plasmodium vivax*

There is a need for better drugs competent for killing parasites in all stages of Plasmodium, such as liver, blood, and mosquito. A single dose of tafenoquine, another 8-aminoquinoline, when taken with chloroquine, has been able to cure patients with *P. vivax*. Tafenoquine was recently approved by the United States Food and Drug Administration (FDA) for the eradication of *P. vivax* under the Krintafel label for individuals with G6PD activity greater than 70%. Tafenoquine has also been approved by the FDA for prophylaxis at various doses, under the Arakoda label, with a similar indication for G6PD deficiency. The single dose regime of tafenoquine, imparts an advantage in reducing non-compliance currently seen with long primaquine dosing schedules. However, tafenoquine, like primaquine is known to cause haemolysis in G6PD-deficient persons (83). Unlike primaquine, tafenoquine has a relatively long half-life of about 14 days. As a result, patients with G6PD deficiency may experience haemolysis for days (84). A companion qualitative diagnostic test is being made available with the drug to be used as a point of care diagnostic by community health workers before administering tafenoquine to the malaria positive. India can consider the deployment of drug after due regulatory considerations (85).

## 10 Drug resistance

Chloroquine is the primary first-line treatment for vivax malaria. The widespread resistance of vivax malaria to chloroquine in many countries in the Middle East, South Asia, Southeast Asia, East Africa and the Americas poses a challenge to control systems and control programs and highlights the critical need to develop new tools aimed specifically at treating *P. vivax* (86–91).

In Papua New Guinea and Indonesia, *P. vivax* was first reported to be resistant to chloroquine in 1989 and has since been identified in 12 countries. This resistance was reported after 30 years of recording chloroquine-resistant *P. falciparum* (86, 92). In 1989, the first documented instances of chloroquine-resistant *P. vivax* were recorded in Papua New Guinea. At that time, high-level chloroquine resistance had become prevalent in areas such as Indonesia and Oceania, which were recognized as epicenters of chloroquine resistance (92). Countries like Indonesia have shifted towards artemisinin-based combination therapies (ACTs) for treatment of *P. vivax* in the light of chloroquine resistance. Atovaquone-proguanil, quinine plus either tetracycline or mefloquine, or doxycycline have been proposed as therapeutic options in areas where *P. vivax* is resistant to chloroquine (49). It is also concerning that the new ACT medication has a lower impact on *P. vivax* than on *P. falciparum* (93). Dihydroartemisinin–piperaquine or Artemether–lumefantrine combinations are generally used, where a high level of resistance of *P. vivax* to chloroquine is detected (78). Therefore, there is an urgent need for revised *P. vivax* treatment guidelines to control and eliminate *P. vivax* malaria.

In India, the treatment of vivax malaria with chloroquine remains the secure and effective method (94, 95). Studies on drug efficacy are being routinely conducted in India to monitor the development of chloroquine resistance in this species (22). However, chloroquine resistance in some places was reported sporadically in the country in 2000 (96, 97). But extensive studies on therapeutic efficacy conducted regularly in India at various sites could not confirm the presence of chloroquine resistance (94, 95, 98).

## 11 Deficiency of glucose-6-phosphate dehydrogenase

G6PD deficiency is known to be associated with protection against malaria and related to *P. vivax* treatment (99). The risk–benefit ratio has changed because compared to the general population, the G6PD prevalence in the patient with infection of *P. vivax* patients is much lower (100). For the primaquine treatment guideline development, an evidence base of the geographical allele distribution of G6PD deficiency in *P. vivax* infected patients is necessary. This information on G6PD deficiency prevalence in any endemic areas provides support for the development of therapeutic strategies and screening for 8-aminoquinoline that reduce the risk of hemolysis caused by consumption of 8-aminoquinoline.

Primaquine can be given without blood testing in countries where there is essentially no G6PD deficiency, such as parts of Latin America and China. However, in India, the overall prevalence has been documented to be 1.9%, with a varied range from 0.8 to 6.3% (101). Its prevalence in the east (6.7%), central (6.1%), and north (5.8%) of India is higher than in the west (4.1%) and south (3.2%), according to a review of 224 studies in India. Scheduled tribes have the highest rate of G6PD deficiency among ethnic groups (5.5 percent) (22). In India, testing of G6PD deficiency facilities are not readily available, though incorporated as a priority for operational research under the National Framework for Malaria Elimination (19).

It is metabolized by the CYP2D6 isozyme of cytochrome P-450 (102). Individuals with exact CYP2D6 polymorphic alleles are unsuccessful in metabolizing primaquine and thus treatment may fail (102). It remains unclear as to what extent CYP2D6 polymorphisms can lead to treatment failure for parasite hypnozoites and later stages of the parasite, but the polymorphism is naturally occurring in the gene. CYP2D6 encoding leads to a wide range of metabolic activity, from above normal to none. Thus, cases of *P. vivax* with significant or no CYP2D6 depletion may relapse even with adequate adherence to good medication. The frequency of altered CYP2D6*10 allele (a specific genetic variant of the CYP2D6 gene) is relatively common (approximately 35%) in Southeast Asians (103). However, the distribution pattern of CYP2D6 alleles among different endemic regions for *P. vivax* is not fully understood. Hence, a better understanding of the CYP2D6–primaquine relationship in the different endemic areas for *P. vivax* will help to determine the failure rates of primaquine and to achieve better overall effectiveness of anti-relapse therapy (104).

## 12 Control of *Plasmodium vivax*

Hypnozoites impose a serious challenge to *P. vivax* elimination. At present, the only method of removing hypnozoites is the use of time-tested primaquine. Hypnozoite elimination was possible at the community level in large military populations where mass drug administration was enforced (105, 106). Vivax malaria elimination in large civilian populations will require some form of mass drug administration with a safe and effective drug.

India’s mosquito population is diverse, with approximately 415 species, including six major regional vectors. *Anopheles culicifacies* and *Anopheles fluviatilis* are the primary malaria vectors, responsible for 75–80% of the disease burden (107). *An.culicifacies* is a zoophilic species that adapts to changing ecological conditions, such as deforestation and population migration. *Anopheles fluviatilis,* a foothill species, shares habitat with *An. culicifacies* and is highly efficient in maintaining year-round malaria transmission. *Anopheles minimus* and *Anopheles baimaii* are highly efficient vectors in the eastern and northeastern regions, contributing to 5% of total cases. *Anopheles stephensi*, an urban vector, is found in metropolitan areas and contributes to about 10% of annual cases. *Anopheles sundaicus*, a brackish water species unique to the Andaman and Nicobar Islands, is primarily invasive and expanding with urbanization. Secondary vectors like *An. annularis, An. subpictus, An. nivipes An. philippinensis, An. maculatus,* and *An. jeyporiensis* also participate in malaria transmission in their areas of distribution. The increasing prevalence of insecticide-resistant malaria vectors in India has raised concerns. *Anopheles culicifacies* has shown resistance to all three insecticides, while other primary malaria vectors display resistance to both DDT and Malathion (108, 109). *Anopheles minimus* exhibits some resilience to DDT but remains susceptible to most other insecticides (110). However, there have been reports of behavioral avoidance in response to certain insecticides (111). To eliminate *P. vivax* malaria, vector control programs must run continuously as concerted anti-transmission measures, like mass distribution of insecticide impregnated nets (LLINs) targeting the local vectors at least for five years preferably beyond.

Moreover, *P. vivax* is a major public health issue in Indian cities. Malaria control in such areas is difficult due to the developmental activities, proliferation of shanties and migratory population. A separate program under the umbrella of national malaria control program namely, “Urban Malaria Scheme” covers these urban areas. The “Urban Malaria Scheme,” officially approved and sanctioned in November 1971, was dedicated to combat malaria in urban areas through a multifaceted approach (112). A fundamental element of this program involved the implementation of urban bylaws, which aimed to prevent mosquito breeding in residential blocks, government and commercial buildings, and construction sites by enforcing specific regulations. Additionally, the scheme introduced larvivorous fish into various water bodies, including streams, lakes, and ornamental ponds, where these fish effectively consumed mosquito larvae, offering a potent method for controlling mosquito populations. In areas where the use of fish was not practical, the scheme employed larvicides to eliminate mosquito larvae. Complementing these measures, awareness campaigns conducted by municipal bodies and urban area authorities played a vital role in educating and engaging the community in the collective effort to prevent and manage urban malaria. The malaria control strategies within this program include administering treatment through passive institutions such as medical facilities, hospitals, private and public sector pharmacies, and private healthcare providers. Dedicated malaria clinics are established in major urban centers, as well as in local government, railways, and defense services. In 2014, out of all the 4% of malaria cases, 12% PV cases were reported from urban areas. In the same year, *P. vivax* prevalence was 98 percent among all malaria cases in the Urban Malaria Scheme (112). Therefore, urban malaria schemes should be strengthened for the control of vectors resulting ultimately in the reduction of malaria cases.

## 13 Genetic diversity of *Plasmodium vivax*

*Plasmodium vivax* is genetically far more varied than *P. falciparum,* with the possibility of many clones present in a single infection, a phenomenon reported in several studies worldwide, even in areas with low-grade transmission (10, 11, 113–117). This scenario is in contrast to *P. falciparum,* the diversity of which is related to the regions of occurrence (118). High genetic variability in *P. vivax* is seen to confer it an ability to evade host immune response, increase drug resistance, and perhaps will be a critical challenge to the vaccine development program against this parasite. It also equips the species with greater adaptability to different anophelines and human hosts. Comparative genomic structure of the population of two species *P. vivax* and *P. falciparum,* showed a less structured population of *P. vivax*, suggesting a higher occurrence of gene flow between different geographic areas (11, 117). The main cause of the higher diversity of *P. vivax* could be due to relapse, which provides greater potential for multiple infections (113, 115). Immunity to *P. vivax* develops faster than *P. falciparum*. Studies are warranted to understand the relationship between genetic and antigenic variability crucial for the vaccine development program against *P. vivax*. Genetic diversity results from cross-mating between different co-existing strains of the parasite which, when passed on from the mosquito to the human hosts, are further propagated (10). Further, gametocyte specific pvs25 gene transcripts of *P. vivax* can be detected with molecular techniques (119). Almost 2,800 genes have been characterized, exclusive to *P. vivax*, possibly as a result of duplication within the *P. vivax* lineage providing it with the advantages mentioned earlier (120). Moreover, *P. vivax* is more antigenically conserved. The dynamics of polymorphism in *P. vivax* parasites need to be studied in order to understand its immune responses and evasion strategies.

## 14 Potential vaccine

In addition to other drugs that target the dormant stage of the liver, the development of vaccines against vivax malaria is of particular importance. A vaccine will protect against clinical illness, reduce transmission and potentially target each stage of the *P. vivax* life cycle. A vaccine against *Plasmodium vivax* (*P. vivax*) holds the potential to significantly reduce malaria transmission by targeting the liver-stage of the parasite, thereby lowering the parasite reservoir in humans and the risk of transmission to mosquitoes. This vaccine can work in conjunction with other malaria control measures, provide long-term protection, break the cycle of recurring infections, reduce the healthcare burden, and offer sustained immunity. It aligns with global health security goals and can yield economic benefits such as increased productivity and reduced healthcare costs. However, developing and deploying a *P. vivax* vaccine, especially in the context of India’s goal for malaria elimination by 2030, faces numerous challenges.The challenges in developing a *P. vivax* vaccine arise from the parasite’s high genetic diversity and complex life cycle. Potential vaccine candidates are categorized into three groups: preerythrocytic (PEVs), blood-stage (BVs), and transmission-blocking vaccines (TBV).

PEVs target sporozoite antigens and aim to prevent hepatocyte invasion and the development of hypnozoites. Promising candidates include circumsporozoite surface protein (CSP), thrombospondin-related anonymous protein (TRAP), and PvCelTOS. A multigenic vaccine combining PvCSP and PvTRAP has shown potential in animal models and is moving towards clinical trials (121, 122).

BVs focus on the asexual blood stage to alleviate malaria symptoms and reduce parasitemia and transmission. Key antigens include PvDBP-II (which interacts with the DARC/Duffy receptor), PvRBP-2b (which targets the TfR1/CD78 receptor), PvAMA-1, and the PvMSPfamily, which are involved in the invasion process. Others were recently described, such as GAMA, PvTRAg (which targets the Band 3 receptor or Basigin/CD147 receptor, such as TRAg-38), MAELB, ETRAMP, RON2 and EBP-2. They are expressed on the surface, microneme or rhoptry of the parasite and inhibit parasite invasion of reticulocytes (123–125).

TBVs target the sexual stages of the parasite in both the mosquito’s midgut and blood. Candidates such as Pvs25, Pvs28, Pvs45, Pvs48, and Pvs230 aim to block transmission by preventing the maturation of sexual phases in the mosquito host (126–130).

The Malaria Vaccine Technology Roadmap, developed by the world’s leading funders for malaria vaccine development, has envisaged the development of vaccines with protective efficacy of at least 75% against clinical malaria against both *P. falciparum* and *P. vivax* to reduce transmission of human malaria and accelerate malaria elimination globally by 2030 (131).

## 15 Challenges for *Plasmodium vivax* malaria elimination

- **Diagnosis:** The low density of *P. vivax* in the blood-stage are common (132, 133), making laboratory diagnosis particularly difficult. Since *P. vivax* usually infects reticulocytes and restricts parasite densities, diagnostic tests with high sensitivity are needed. Skilled microscopy is relied upon for blood smear-based diagnosis, but its use may be challenging in resource-poor settings. It has been observed that in certain areas, *P. vivax* infections, around 70%, were found to be below the detection limit by microscopy, posing an additional challenge to elimination efforts (133). Furthermore, sub-microscopic infections found in all settings seem to have greater comparative importance in low-incidence areas for achieving and maintaining transmission elimination.
- **Presence of hypnozoite stage:** *P. vivax* has a dormant hypnozoite stage in the liver, and limited treatment options are currently available. Despite scientific advancements, technology to recognize or detect the presence of hypnozoite is lacking (134).
- **G6PD deficiency:** Additionally, primaquine drug, which targets hypnozoites can potentially cause haemolysis in individuals who are G6PD deficient. Thus, testing for G6PD deficiency is desirable before the administration of primaquine (135). The testing facilities are not rampantly available in Indian public health settings. The 14 days treatment course of primaquine, with uncertain compliance and prevalence of G6PD deficient cases with the extensive genetic disorder, could cause severe haemolyticanaemia in approximately 8% of the people in a malaria endemic country (136).
- **Genetic diversity and resistance to chloroquine**: The failure of primaquine against latent *P. vivax* in individuals with damaged P450 2D6 alleles increases the substantial pool of primaquine ineligibles (137). When there is an acute natural polymorphism in the gene encoding CYP2D6, it results in various metabolic activities that confer chloroquine resistance (138). Also, acute regular polymorphisms in the CYP2D6 gene result in different metabolic activities that confer chloroquine resistance. As a result, many Asians may not be capable to fully metabolise the primaquine and achieve successful radical treatment (139). Alternative therapies such as ACTs are rarely assessed as a companion drug to primaquine in the crucial radical cure where *P. vivax* malaria is common. Hence alternate anti-relapse modalities and enabling strategies are needed to overcome this limitation to achieve *P. vivax* elimination.
- **Outdoor transmission:** Several vectors of *P. vivax* species exist in South-East Asia and South Asia, where >90% of infections occur. They flourish in diverse habitats, with adults preferring to rest indoors and outdoors and demonstrate a broad range of breeding and feeding behavior (140). Insecticide-impregnated bed nets and indoor residual spraying have been the primary malaria interventions. While bed nets and IRS provide high levels of protection indoors, no cost-effective, practicable, or scalable vector control techniques have been identified to provide similar levels of protection against outdoor biting mosquitoes.

## 16 Conclusion

There have been major gains against malaria, including *P. vivax,* globally over the last decade. However, there exist significant challenges for control of *P. vivax*, and its elimination may not be achievable with current malaria control tools. This parasite species has adapted to several vector species across varied ecologically diverse habitats and regions. *P. vivax* has a complex biology; it is difficult to detect the low parasite densities in infected individuals, the survival of hypnozoites causes relapses with polymorphism, and the appearance of gametocytes early in the course of infection all of which aid in infection transmission. Better *P. vivax* diagnostic tools are needed, particularly point-of-care rapid diagnostic tools with high sensitivity and specificity. Highly sensitive tests for detection of asymptomatic infection of *P. vivax*, and reliable tests for G6PD deficiency prior to dosing primaquine, may aid in better control and eventually elimination. New drugs that do not cause haemolysis are needed to replace primaquine, and anti-relapse therapeutics with shorter treatment regimens for better compliance, would greatly aid elimination efforts. As the world looks forward to malaria elimination, an effective surveillance system should be in place for monitoring, evaluation and evidence generation as the key interventions are rolled out and program are metamorphosed from control to achieve elimination goals. A multistage vaccine to prevent *P. vivax* infection and transmission would be a welcome development to achieve and sustain the elimination of malaria.

## Author contributions

PPS and AK majorly drafted and edited different versions of the manuscript. SS, KH, ST, MR, and PPS significantly contributed with graphics, literature, provided technical inputs to the manuscripts for improvement. All authors contributed to the article and approved the submitted version.
